# Increased Cell Wall Teichoic Acid Production and D-alanylation Are Common Phenotypes among Daptomycin-Resistant Methicillin-Resistant *Staphylococcus aureus* (MRSA) Clinical Isolates

**DOI:** 10.1371/journal.pone.0067398

**Published:** 2013-06-13

**Authors:** Ute Bertsche, Soo-Jin Yang, Daniel Kuehner, Stefanie Wanner, Nagendra N. Mishra, Tobias Roth, Mulugeta Nega, Alexander Schneider, Christoph Mayer, Timo Grau, Arnold S. Bayer, Christopher Weidenmaier

**Affiliations:** 1 Interfakultäres Institut für Mikrobiologie und Infektionsmedizin, Microbial Genetics, University of Tübingen, Tübingen, Germany; 2 Division of Infectious Diseases, LA Biomedical Research Institute at Harbor-UCLA Medical Center, Torrance, California, United States of America; 3 David Geffen School of Medicine at UCLA, Los Angeles, California, United States of America; 4 Cecolabs UG, Tübingen, Germany; 5 Interfakultäres Institut für Mikrobiologie und Infektionsmedizin, University of Tübingen, Tübingen, Germany; 6 Interfakultäres Institut für Mikrobiologie und Infektionsmedizin, Microbiology/Biotechnology, University of Tübingen, Tübingen, Germany; University Medical Center Utrecht, The Netherlands

## Abstract

Multiple mechanisms have been correlated with daptomycin-resistance (**DAP-R**) in *Staphylococcus aureus*. However, one common phenotype observed in many DAP-R S*. Aureus* strains is a thickened cell wall (CW). The first evidence for an impact of CW-linked glycopolymers on this phenotype was recently demonstrated in a single, well-characterized DAP-R methicillin-susceptible *S. aureus* (**MSSA**) strain. In this isolate the thickened CW phenotype was linked to an increased production and D-alanylation of wall teichoic acids (WTA). In the current report, we extended these observations to methicillin-resistant daptomycin-sensitive/daptomyin-resistant (**DAP-S/DAP-R**) strain-pairs. These pairs included methicillin-resistant *S. aureus* (**MRSA**) isolates with and without single nucleotide polymorphisms (SNPs) in *mprF* (a genetic locus linked to DAP-R phenotype). We found increased CW dry mass in all DAP-R vs DAP-S isolates. This correlated with an increased expression of the WTA biosynthesis gene *tagA*, as well as an increased amount of WTA in the DAP-R vs DAP-S isolates. In addition, all DAP-R isolates showed a higher proportion of WTA D-alanylation vs their corresponding DAP-S isolate. We also detected an increased positive surface charge amongst the DAP-R strains (presumably related to the enhanced D-alanylation). In comparing the detailed CW composition of all isolate pairs, substantive differences were only detected in one DAP-S/DAP-R pair. The thickened CW phenotype, together with an increased surface charge most likely contributes to either: i) a charge-dependent repulsion of calcium complexed-DAP; and/or ii) steric-limited access of DAP to the bacterial cell envelope target. Taken together well-defined perturbations of CW structural and functional metrics contribute to the DAP-R phenotype and are common phenotypes in DAP-R S*. Aureus* isolates, both MSSA and MRSA.

**Note:** Although “daptomycin-nonsusceptibility” is the generally accepted terminology, we have utilized the term “daptomycin resistance” for ease of presentation in this manuscript

## Introduction

The rising number of multi-antibiotic-resistant strains has seriously limited the treatment options in severe *S. aureus* infections (e.g. MRSA; VISA) [1,2]. In this regard, daptomycin (**DAP**) has become one of the most important therapeutic agents [3,4]. The recent emergence of DAP-resistant (**DAP-R**) strains, associated with clinical treatment failures [5–7], has spiked an interest in determining the molecular bases of DAP-R. Interestingly, DAP-R can be linked to several distinct, and perhaps, unrelated mechanisms, and is often multifactorial. In a number of DAP-R isolates, expression of genes that are involved in maintenance of the bacterial surface positive charge (e.g., *dltA-D*; *mprF*) is perturbed, usually translating into “gain-in-function” phenotypes [8–10]. The phenotypic readout of such gains-in-function has been enhanced positive envelope surface charge, presumably creating a “charge-repulsive milieu”, mitigating calcium-DAP: cell membrane (CM) interactions [11]. In addition, DAP-induced changes in CM permeabilization [12], as well as alterations in CM biophysical order (resulting in extremes of CM fluidity or rigidity) have also been observed in relation to the DAP-R phenotype [13]. Although not a universal association [14], the most frequently described genetic mutations observed in DAP-R S*. aureus* strains are single point mutations (**SNPs**) in various regions of the *mprF* open reading frame, with or without additional point mutations in the *yyc* operon [11,14–16]. MprF is responsible for the lysinylation of phosphatidylglycerol (**PG**) [17] and flips the positively-charged product, lysyl-PG (**L-PG**) to the outer CM leaflet [18]. The *yyc* operon encodes for the YycFG (WalKR) two-component regulatory system, which is believed to regulate fatty acid biosynthesis [19] and also to modulate general CW homeostasis to a variety of stressors [20].

Of interest, in many, but not all, DAP-R S*. aureus* strains, a thickened CW phenotype has been documented by electron microscopy [8,9,13]. In this regard, our labs have recently provided the first evidence that this thickened CW phenotype is linked to an increased expression of wall teichoic acid (WTA) biosynthesis genes (*tag*), in a single, well-characterized methicillin-susceptible *S. aureus* (MSSA) DAP-R isolate [21]. WTA biosynthesis is a complicated process (Figure S1), starting with synthesis of a disaccharide linkage unit, which requires the enzymes TagO and TagA [22,23]. These enzymes transfer GlcNAc-1-phosphate and ManNAc, respectively, from UDP-activated precursor molecules to undecaprenyl-phosphate (C_55_–P). The repeating units are then incorporated by several priming and polymerizing enzymes, and after biosynthesis is completed, the repeating units are modified with D-alanine [23]. The *dltABCD* operon encodes the required enzymes, and is therefore responsible for the modulation of the net charge of the teichoic acid polymers [24]. The enhanced expression of the *tagA* gene in the single DAP-R MSSA strain noted above correlated with elevated WTA production; this DAP-R strain also demonstrated increased *dltA* expression, which was associated with augmentation in the proportionality of WTA D-alanylation. On the other hand no significant changes in CW peptidoglycan cross-linkage or in the O-acetylation profiles (as had been previously reported for other DAP-R strains [15]) were found in this DAP-R MSSA strain.

In the current report, we expand upon the preliminary report above [21] by: i) investigating WTA production and D-alanylation profiles in a cadre of DAP-S/DAP-R MRSA strain-pairs; ii) studying DAP-R strains, both with and without *mprF* SNPs; and iii) utilizing advanced HPLC techniques to adjudicate comparative CW muropeptide compositional analyses of the DAP-S/DAP-R isolate-pairs.

## Material and Methods

### Bacterial strains

The four DAP-S/DAP-R MRSA study pairs used in this investigation were clinical bloodstream isolates from the Cubist Pharmaceuticals isolate collection (courtesy of Dr. Aileen Rubio; Lexington, MA). This strain-set was prioritized for the current study because it has been previously well-characterized in terms of: i) strain-pair isogenicity [14]; ii) antimicrobial peptide cross-resistances [14]; iii) CM metrics [14]; and iv) demonstration of a thickened CW phenotype among the DAP-R isolates [14]. As previously documented, the DAP-S and DAP-R isolates within a strain-pair were isogenic on the basis of PFGE analysis, *agr* typing, *spa* typing, inferred clonal complex typing and SCC*mec* typing [14]. The DAP-R isolates of the CB5021-CB5020 (resistant) and CB5062-CB5063 (resistant) pairs contain no *mprF* or *yyc* operon SNPs, whereas the DAP-R strain of the CB1663/CB1664 strain-pair carries single point mutations in both *mprF* and *yycG* that lead to amino acid exchanges L826F in MprF and R86H in YycG, respectively [14]. The genotyping and SNP data have been previously reported [14]. The strain-pair, CB5088/CB5089 exhibits no CW thickening in the DAP-R strain, and was included as a control. Strain CB5089 contains a point mutation that leads to the amino acid exchange S295L in MprF.The daptomycin MICs and SNP characteristics are listed in Table **1**. These data have been previously reported [14]

**Table 1:** Bacterial strains.

	MICs and mutations in MprF/YycG		
	Daptomycin MIC [µg/ml] [14]	Amino acid change in MprF [14]	Amino acid change in YycG [14]
CB1663 (Dap-S)	0.5	L826F	R86H
CB1664 (Dap-R)	4		
CB5021 (Dap-S)	0.25	none	none
CB5020 (Dap-R)	1		
CB5062 (Dap-S)	0.5	none	none
CB5063 (Dap-R)	8		
CB5088 (Dap-S)	0.5	S295L	none
CB5089 (Dap-R)	2-4		

### Wall teichoic acid (WTA) isolation and purification

We isolated CW and WTA specifically as described in detail before [24,25]. In brief, bacteria were cultivated overnight in B-Medium (1% peptone, 0.5% yeast extract, 0.1% glucose, 0.5% NaCl and 0.1% K_2_HPO_4_) containing 0.25% (wt/vol) glucose, washed twice in sodium acetate buffer (20 mM, pH 4.7) and disrupted in the same buffer with glass beads for 1h on ice in a cell disruptor (Euler). We determined the total amount of protein-free CW contained within our strain-sets by weighing the CW preparation after drying. The CW dry weight determinations were derived from 5 independent isolations. To allow better strain to strain comparability cell wall dry weight was expressed as mg cell wall dry weight per g cell wall wet weight. In parallel, WTA was released from purified CWs by treatment with 5% trichloroacetic acid in sodium acetate buffer for 4 h at 60°C. CWs were removed by centrifugation. WTA was quantified by determining its inorganic phosphate (Pi) content as described [25]. The isolation was performed in triplicate for each strain, and assayed in triplicate for their respective Pi content.

### Quantification of D-alanine content

D-alanylation of the WTA polymers was assayed and quantified as described before [21,26]. In brief, D-alanine esters were hydrolyzed by a mild alkaline hydrolysis carried out at 37°C for 1 h in 0.1 M NaOH. The supernatant was neutralized, dried under vacuum, and used for precolumn derivatization with Marfey’s reagent (1-fluoro-2, 4-dinitrophenyl-5-L-alanine amide; Sigma). Amino acid derivates (detection at 340 nm) were then separated as described before [21] and analyzed with the ChemStation software. Data were expressed as percent of WTA (± SD) that was D-alanylated. A minimum of three independent runs was performed.

### Quantification of *dlt* and *tagA* expression

We examined the relationship between WTA production and D-alanylation profiles with gene expression related to these two phenotypes (*tagA* and *dltA*, respectively) [21,27,28]. For RNA sample preparation, fresh overnight cultures of *S. aureus* strains were used to inoculate TSB to an optical density at 600 nm of 0.1. Cells were harvested during both exponential and stationary growth phases. Total RNA was isolated from the cell pellets by using the RNeasy kit (Qiagen, Valencia, CA) and the FASTPREP FP120 instrument (BIO 101, Vista, CA), according to the manufacturer’s recommended protocols.

Primers to amplify *dltA* were dlt-F-1 and dlt-R [8,21]. Primers for *tagA* were tagA-F and tagA-R [21]. All RT-PCR experiments were performed in triplicate, with the *gyrB* gene expression used as a control and baseline for fold-changes in expression of *tagA* and *dltA*.

### Surface charge assays

We determined the relative surface charge with a cytochrome *c* binding assay as described previously [24]. BHI broth overnight cultures were washed with 20 mM MOPS buffer (pH 7.0) and then resuspended in the same buffer at OD_578_ = 1.0. Cells were incubated with 0.5 mg/ml cytochrome *c* for 10 min, and the amount of cytochrome *c* remaining in the supernatant was determined spectrophotometrically at OD_530_ nm. The more unbound cytochrome *c* was detected in the supernatant, the more relative positive charge on the bacterial surface. Data were expressed as mean (± SD) amount of unbound cytochrome *c*. At least three independent runs were performed on separate days.

### Muropeptide analysis by HPLC

All strains were grown in Mueller-Hinton broth to an OD_578_ = 0.7 or for 24 hrs. If indicated, 0.7 g/l glycine or alanine was added. The CW of the study strains was isolated, then digested with a muramidase, and analyzed via HPLC essentially as described before [29] (Cecolabs; Tuebingen, Germany). The analyses were done on an Agilent 1200 system with a Prontosil C18-RP column (Bischoff Chromatography, Leonberg, Germany).

### Mass spectrometry (MS) analysis

HPLC peaks-of-interest from the muropeptide analysis were collected and analyzed by LC-MS. The liquid chromatography system used was a Dionex Ultimate 300 RS coupled to a BrukermicrOTOF II set on positive ion mode. CW components were separated on a Phenomenex Gemini 150 x 4.6 mm C18 110Å 5µM column (Phenomenex, Aschaffenburg, Germany). The 45 min program was run with a flow rate of 0.2 ml/min and 0.1% formic acid with 0.05% ammonium formate as buffer A and 100% acetonitrile as buffer B. After a 5 min washing step with 100% buffer A, a 30 min linear gradient to 40% buffer B followed. A 5 min gradient delay and 5 min of re-equilibration completed the method. The injection volume of the single peaks was 20 µl.

### Amino acid analysis

The CW of the strain-pair, CB1663 and CB1664, was isolated after 24 h in triplicate (see above). The lyophilized CW was hydrolyzed by adding 600 µl 6N HCl to 45 mg substrate and incubated by 110°C for at least 18 h. The released amino acids were then neutralized for 72 h in NaOH atmosphere under vacuum conditions. OPA derivatization was performed in the injection-needle of the HPLC as pre-column derivatization. Ortho-Phthaldialdehyde (OPA) was purchased from GRACE, Davison (Lokeren, Belgium). The stock solution of 10 mg/ml was diluted 1:10 in 1 M Borate-buffer (61.8 g borate in 1 liter of HPLC-grade-water). 6 µl OPA and 1.5 µl substrate were mixed for 90 sec in the injection needle and then separated via HPLC with an Agilent 1200 series HPLC-system using a Grom-SIL OPA-3 (5µm) 4.0 x 150 mm column. The gradient was run in 24 min from 100% buffer A (25 mM Sodium-phosphate buffer with pH =7.2) to 100% buffer B (50% 25 mM sodium-phosphate buffer, pH =7.2, 35% methanol, and 15% acetonitrile) in a stepwise manner. The column temperature was 25°C and the flow rate was 1.1 ml/min. The detector was set on fluorescence with 330 nm excitation and 450 nm emission. The data was analyzed with the ChemStation software.

### Statistical analysis

Statistical analyses were performed with Graphpad Prism®, using appropriate statistical methods as indicated. *P* values ≤ 0.05 were considered as significant.

## Results

### Total CW and WTA content

We detected significant differences in the amount of total CW produced between the DAP-S vs DAP-R isolates within each strain-pair (Table 2). For example, the ratio of mg CW dry weight/g of CW wet weight in the DAP-S strain CB5062 was 7.5 (± 5.7) vs. DAP-R strain CB5063 at 18.2 (± 9.9) (p < 0.05).

**Table 2:** WTA and WTA D-alanylation in the DAP-S/DAP-R strain pairs.

	Cell wall (CW) dry mass and WTA amount		
	CW mass [mg dry weight/g wet weight]	Amount of WTA [nmol Pi/mg CW]	Amount of WTA D-alanylation [% nmol D-alanine/nmol Pi]
CB1663	12.6 ± 4.2	93.4 ± 24.0	45.2 ± 4.8
CB1664	21.8. ± 6.6*	144.6 ± 22.0*	82.1 ± 16.7*
CB5021	12.9 ± 5.9	87.7 ± 37.2	32.9 ± 5.8
CB5020	25.3 ± 10.3*	175.3 ± 42.7*	53.7 ± 9.3*
CB5062	7.5 ± 5.7	67.4 ± 18.3	32.8 ± 8.8
CB5063	18.2 ± 9.9*	169.5 ± 70.5*	57.7 ± 18.8*
CB5088	10.7 ± 2.6	99.1 ± 50.1	41.4 ± 15.4
CB5089	9.2 ± 3.0^ns^	106.5 ± 54.1^ns^	38.8 ± 14.7 ^ns^

Dry mass of CW was quantified as [mg dry weight/ g wet weight] n 5 The amount of WTA was determined by a colorimetric assay and expressed as [nmol Pi/mg cell wall] n 4 (except 5062/5063). The rate of D-alanylation of WTA repeating units was determined by HPLC n 3 Statistical analysis was performed by Student’s t-test (except CB5062/5063 Welch corrected t-test). Significance: *p-value <0.05 vs. DAP-S strain;

In addition, there were significant differences in the amount of WTA found in the CWs of each strain-pair in which the DAP-R strain exhibited the thickened CW phenotype, with DAP-R strains producing significantly more WTA than their respective DAP-S strains. In contrast, in the strain-pair CB5088/5089 in which the CWs were of equivalent thickness, neither CW dry weight nor WTA amount was significantly different (Table 2).

### WTA D-alanylation

In addition to the significant increases in overall WTA content in the DAP-R strains above, there were also substantial differences in the proportion of WTA that was D-alanylated when comparing the DAP-R vs DAP-S isolates. The percentage of D-alanine contained within the WTA (nmol D-alanine/nmol Pi) in all DAP-R strains was significantly higher than that observed in their respective DAP-S parental strains. In the control strain pair, CB5088/CB5089, without differences in CW thickness, no differences in D-alanylation of WTA were detected (Table 2).

### Gene expression analysis

**Figure 1:**
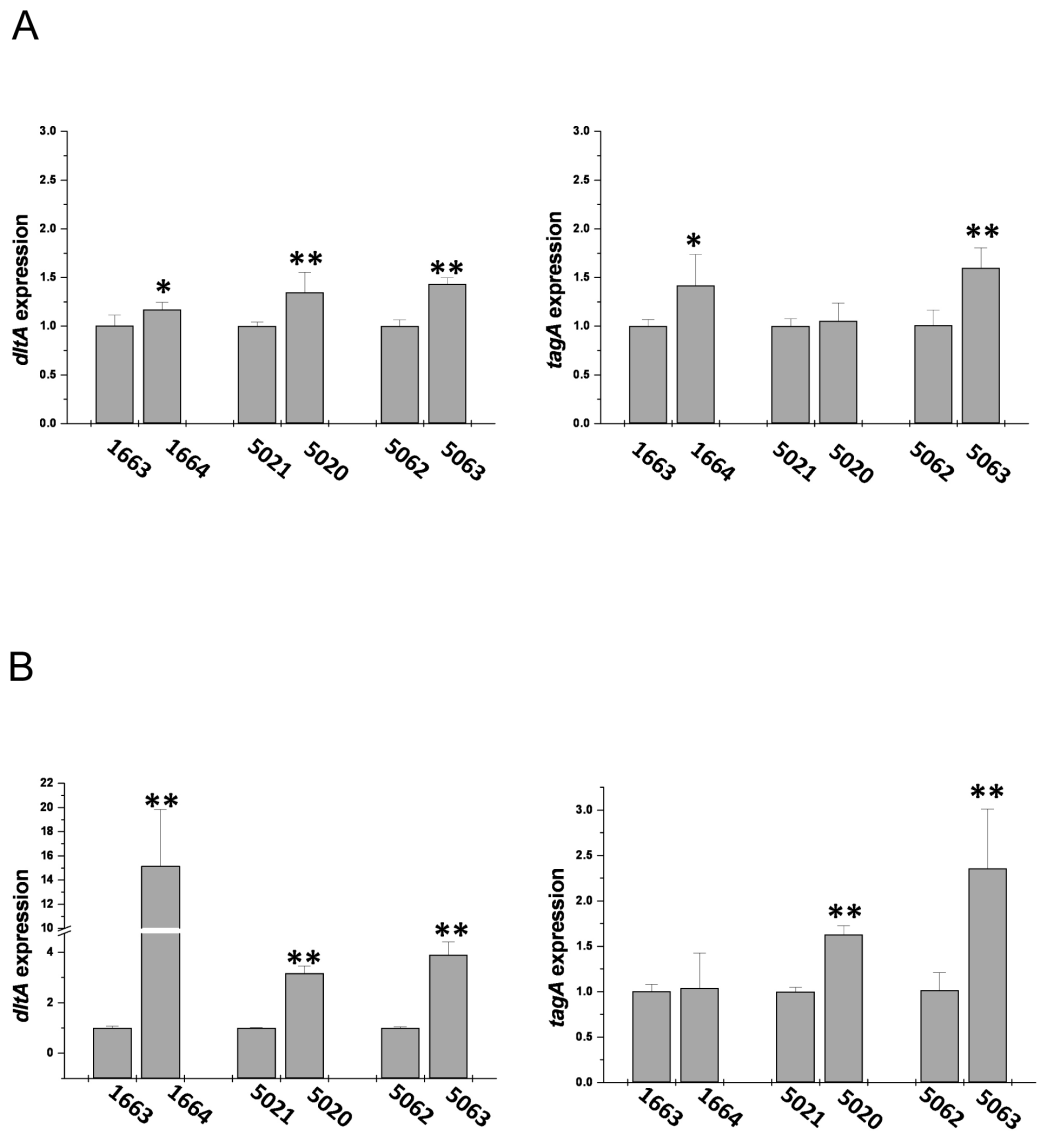
Expression profiles of *dltA* and *tagA*. Expression in exponential (**A**) and stationary growth phase (**B**). Values from exponential and stationary growth phase RNA samples were normalized vs. housekeeping gene, *gyrB*, expression levels; data from the DAP-S strains were set to 1 to allow comparison of data from different samples with their respective DAP-R isolates. *P < 0.05 and **P < 0.001.

As shown in Figure **1**, during exponential growth phase, in all three of the strain-pairs, *dltA* expression was significantly greater in the DAP-R isolate as compared to the respective DAP-S parental strain. A similar outcome was observed for *tagA*, with expression of this gene being significantly higher in two of the DAP-R isolates as compared to their respective DAP-S parental strains. This pattern of differential expression between the DAP-S/DAP-R strain pairs was even more notable during stationary phase of growth. For *dltA*, all three DAP-R strains exhibited substantially higher expression than their respective DAP-S parental strains. Moreover, for *tagA*, all three DAP-R strains exhibited increased expression as compared to their DAP-S parental strains, reaching statistical significance in two of the three comparisons. It should be pointed out that the overall level of expression of both *tagA* and *dltA* was substantially higher during exponential as compared to stationary growth phases. In the control strain-pair (CB5088/CB5089) there were no differences in *tagA* or *dltA* expression levels noted (Figure S2).

### Surface charge

We tested all 3 DAP-S/DAP-R study pairs that exhibited differences in their D-alanine contents. In all three strain-pairs, the DAP-R isolate exhibited significantly more relative positive surface charge vs. its respective parental DAP-S parental strain (Table 3).

**Table 3:** Relative surface charge of DAP-S/DAP-R strain sets.

Strains	% unbound cytochrome *c* in supernatant
CB1663	66 ± 3
CB1664	77 ± 5^*^
CB5021	64 ± 4
CB5020	75 ± 2^*^
CB5062	70 ± 3
CB5063	77 ± 0^ns^

Relative surface charge is expressed as % cytochrome *c* remaining in the supernatant after incubation. The more unbound cytochrome *c* was detected in the supernatant, the more relative positive charge on the bacterial surface. Data were expressed as mean amount of unbound cytochrome *c*. n 3 Statistical analysis was performed by Student’s t-test. Significance: ^*^p-value <0.05 vs. DAP-S strain; p-value CB5062 vs. Cb5063 =0.07

### Muropeptide analysis and calculation of cross-linkage

In order to determine whether there were structural differences in the CW of the DAP-S vs. respective DAP-R strains, the peptidoglycan was isolated, digested into muropeptides and analyzed by HPLC (Figure **S3**). We determined the distribution of monomeric, dimeric, trimeric, and oligomeric muropeptides, and calculated the amount of cross-linkage for each strain (Table 4). For the CB5021/CB5020, CB5062/CB5063, and CB5088/CB5089 strain-pairs, no reduction in cross-linkage was detected, and therefore, no increase in the monomeric, dimeric, and trimeric muropeptides. In contrast, strain pair CB1663/CB1664 showed a significant reduction in cross-linkage (73.8 ± 2.4 vs 65.5 ± 1.5, p = 0.0011), and a concomitantly significant increase in monomers (8.8 ± 1.7 vs 15.7 ± 1.0, p = 0.0005), dimers (14.9 ± 2.1 vs. 18.7 ± 1.4, p = 0.0314), and trimers (9.8 ± 1.3 vs. 11.9 ± 0.9, p = 0.0025). In addition, in DAP-R isolate CB1664, a notable increase of monomeric muropeptide species was seen (only present in very small amounts in CB1663) (Figure **S3**, structures are depicted in Figure **S4**). In contrast, several muropeptide peaks were slightly reduced in DAP-R strain CB1664 vs. DAP-S strain CB1663.

**Table 4:** Distribution of muropeptides and amount of cross-linkage.

	% muropeptides				
Strain-pair	OD_578_=0.7				
	Monomers	Dimers	Trimers	Multimers	Cross-Linkage
CB1663	8.8 ± 1.7	14.9 ± 2.1	9.8 ± 1.3	66.4 ± 4.4	73.8 ± 2.4
CB1664	15.7 ± 1.0***	18.7 ± 1.4*	11.9 ± 0.9*	53.6 ± 2.5**	65.5 ± 1.5**
CB5021	10.1 ± 2.2	16.6 ± 0.5	10.7 ± 0.8	62.6 ± 1.9	71.7 ± 1.9
CB5020	10.4 ± 1.3^ns^	16.7 ± 1.9^ns^	10.9 ± 1.9^ns^	62.0 ± 4.8^ns^	71.4 ± 2.3^ns^
CB5062	10.4 ± 1.3	16.7 ± 0.9	10.9 ± 1.9	62.0 ± 4.8	71.4 ± 2.3
CB5063	10.1 ± 2.2^ns^	16.6 ± 0.5^ns^	10.7 ± 0.8^ns^	62.6 ± 1.9^ns^	71.7 ± 1.8^ns^
CB5088	10.5 ± 2.2	17.7 ± 1.8	11.6 ± 0.6	60.3 ± 4.2	70.8 ± 2.6
CB5089	11.0 ± 2.0^ns^	17.2 ± 1.5^ns^	11.3 ± 0.6^ns^	60.6 ± 3.5^ns^	70.6 ± 2.2^ns^

The numbers give the sum of the peak areas of the indicated fraction. It is the mean value of three different analyses. Cross-linkage was calculated as described [38]: 0.5 x dimers (%) + 0.67 x trimers (%) + 0.9 x oligomers (%). For most strains, there was only a slight or no reduction in cross-linkage observed. The strain pair that shows a reduction (CB1663/CB1664) is highlighted in grey. n 3 Statistical analysis was performed by Student’s t-test. Significance: *p-value <0.05 vs parental strain; **p-value < 0.001 vs parental strain; ***p-value < 0.0001 vs parental strain.

A recent publication indicated that structural changes in the peptidoglycan of *S. aureus* can depend on the available nutrients [30]. We, therefore, analyzed the muropeptide pattern of strain set CB1663/CB1664 at different growth time-points, and tested whether the addition of glycine or alanine to the medium had any effect (Figure **S5**). Only this single strain-pair was analyzed because of its obvious differences in the muropeptide composition between the DAP-S and DAP-R isolates and the significant reduction in cross-linkage with a concomitant doubling in monomeric muropeptides. These differences were not seen in the other three strain pairs. The peaks of strain CB1663 and CB1664 without the addition of extra amino acids were collected and analyzed by mass spectrometry (MS) and the percentage of each muropeptide was calculated (Table **S1**). An overview on muropeptide structures is given in Figure **S4**. At OD_578_=0.7, there was an increase of 4.5 fold in peak 3 (Penta-(Gln)) and 3.9 fold in peak 5 (Penta(Gln) Gly) of DAP-R strain CB1664 vs DAP-S strain CB1663, while peak 11 (the cyclic dimer) was diminished by 50%. While the addition of alanine had no obvious effect on the muropeptide patterns of either CB1663 or CB1664, we saw an 1.9 fold increase in peak 4 (Tetra(Gln) Gly_6_ to Tetra(Gln) Gly_9_) of the DAP-S strain CB1663 when glycine was added. After 24 hrs of growth, the muropeptide pattern of the DAP-R strain, CB1664, exhibited a very strong increase in two monomeric muropeptides (peaks 3 (9.8 fold), and 5 (4.8 fold) and four new monomeric peaks (peak 1 (Tetra(Gln) AlaGly), peak 2 (Tetra(Gln), peak 7 (Penta(Gln) Ala) and peak 8 (acetylated Penta(Gln) Ala)) appeared. Again, there was a decrease in the cyclic dimeric peak (peak 11) by 66%. Peaks 3 and 7 were almost completely lost when glycine was added to the growth medium of strain CB1664. However, alanine had no effect on the muropeptide patterns of either strain. For both strains, the relative percentage of each muropeptide also differed between OD_578_=0.7 and the 24 hr time-point, but to a lesser extent than the differences between the DAP-S and the DAP-R strain when compared at the same harvesting point (Table **S1**).

Since the MS data suggested an increase of muropeptides that contained an alanine within the interpeptide bridge, we analyzed the amino acid composition of the whole cell wall of strains CB1663 and CB1664 after 24 hrs of incubation. In strain CB1664, the amounts of glycine, alanine, and lysine were ~twice as high as in strain CB1663 (Table **S2**).

## Discussion

In *S. aureus*, there is growing evidence for the involvement of CW in the development of the DAP-R phenotype [11]. Several studies have shown that DAP-R S*. aureus* isolates derived from both *in vitro* passage selection, as well as from patients treated with failing regimens of DAP, exhibited significantly thicker CWs as compared to their respective DAP-S parental strain [13,31,32]. This thickened CW phenotype is very reminiscent of that described for VISA isolates [9]; many (but not all) of these DAP-R strains with thickened CWs were, in fact, isolated from patients previously treated with vancomycin [31,32]. These data argue for common molecular mechanisms between the thickened CW phenotype induced by vancomycin and DAP. We recently provided the first evidence for a link between the thickened CW phenotype and an increased production and D-alanylation of WTA. However this study only included a single, well-characterized DAP-R MSSA strain. Since staphylococcal isolates can differ substantially in their phenotypes due to their genetic variability, we extended our observations in the recent study to now include DAP-R MRSA strains.

Regulation of CW biosynthesis is very complex process, and the physiological stress imposed by antibiotic treatment can lead to massive changes in pathways responsible for CW biosynthesis. For example, a gene belonging to the CW stress stimulon, *cwrA* (cell wall–responsive antibiotics; SA2343), was found to be both highly upregulated in several clinical VISA strains [33] and also upregulated upon DAP challenge [34]. However, the complete regulatory mechanisms underlying the VISA and DAP-R phenotypes remain largely elusive, and are most likely multifactorial. For example, Yang et al. [9] confirmed amongst non-VISA, that DAP-R S*. aureus* strains often, but not universally, display thickened CWs (~50% frequency). In contrast, Boyle-Vavra et al. found neither a thick CW phenotype in one DAP-R isolate, nor sequence or transcriptional profiling differences between this DAP-S/DAP-R clinical strain-pair in terms of genes involved in CW metabolism [35]. Therefore, we also included a DAP-S/DAP-R strain-pair that did not show differences in CW thickness as relevant controls. Furthermore, Muthaiyan et al. [34] investigated the transcriptional activation profile of *in vitro* DAP-exposed *S. aureus* cells. They observed that, in addition to inducing genes consistent with CM depolarization, a number of genes involved in the CW stress stimulon were also impacted by *in vitro* DAP exposures. Interestingly, when the transcriptomic inductioprofiles of DAP vs vancomycin vs oxacillin were compared, a large consensus cadre of genes involved in CW synthesis were induced by all three agents (including *vraSR, murAB, pbpB, tcaA* and the various *tag* genes). Thus, DAP can clearly induce the CW stress stimulon in a manner similar to classical CW-active agents. Fischer et al. [36] recently confirmed some of these observations in comparing the transcriptomic and proteomic profiles of a DAP-S/DAP-R MSSA strain-pair. These investigators found a number of genes involved in CW metabolism were up-regulated in the DAP-R isolate, including the WTA biosynthesis enzymes *tagA* and *tagG*, among others.

In the DAP-S/DAP-R strain-pairs in which the DAP-R isolate demonstrated a thickened CW phenotype, the DAP-R strains all showed notable increases in terms of CW dry mass. In turn, this phenotype was likely explicable, at least in part, by the increased amount of CW-attached WTA found in these same DAP-R strains as compared to their respective parental DAP-S isolates. In addition, all DAP-R isolates exhibited a higher percentage of WTA D-alanylation when compared to their DAP-S parental isolates. The control strain-pair CB5088/CB5089 (without CW thickness differences) showed neither differences in WTA amount nor in WTA D-alanylation. This thickened CW phenotype, together with the documented increased positive surface envelope charge amongst the DAP-R strains (presumably related to the enhanced D-alanylation) most likely contributes to either: i) a charge-dependent repulsion milieu, limiting calcium-complexed DAP’s interaction with the bacterial surface; and/or ii) steric-limited access of DAP due to a physically denser CW. It should be pointed out that the above CW perturbations were demonstrated in all three DAP-S/DAP-R strain pairs, irrespective of the presence or absence of SNPs within *mprF* and *yycG*. This suggests that the contribution of perturbations in these gene loci are independent of, and additive to, those involved in the modified CW parameters noted above. This is consistent with the CM (not CW) specificity of these latter two genes which is also underlined by the fact that the altered MprF in the strain CB5089 does not lead to any changes in cell wall composition. MprF is responsible for the lysinylation of CM phosphotidylglycerol, which generates the positively-charged CM phospholipid, L-PG [17,18]. In addition to this synthetic function, MprF is also involved in the inner-to-outer CM flipping of L-PG [18]. On the other hand, the *yyc* operon is involved in the CM stress stimulon and fatty acid metabolism [19].

We have previously compared relevant gene expression profiles in DAP-R vs respective DAP-S strain pairs. For example, for the *mprF* gene, one of two expression profiles distinguish the DAP-R vs DAP-S pairs: i) increased expression during exponential growth (point of expected maximal expression of this gene); and/or ii) unexpected retention of expression during stationary phase of growth [8,10]. In the current study, we saw similar outcome patterns for both *tagA* and *dltA* expression, i.e., i) increased *dltA* expression at both exponential and stationary phases of growth for two of the three DAP-R isolates vs their respective DAP-S parental strains; and ii) unexpected enhancement of *dltA* expression during stationary growth for the remaining DAP-R isolate. A very similar pattern of increased expression profiles was noted for *tagA*, i.e. a substantially increased level of expression at both exponential and/or stationary growth phases. These data speak to a notable “deregulation” of these two operons which are critically responsible for the target CW phenotypes investigated in this study amongst DAP-R isolates: WTA production and D-alanylation of WTA. The genetic network perturbations responsible for this deregulation are under active investigation in our laboratories.

When we investigated the peptidoglycan composition to rule out additional CW perturbations in the strain sets, we could not detect any major changes in the CW composition of these strain-pairs, with the exception of strain-pair CB1663/CB1664. For CB1664 we saw a significant reduction in cross-linkage, and a concomitant increase in the monomeric, dimeric, and trimeric muropeptide content. The increase in some monomeric muropeptides seen at OD_578_=0.7 shows, that the remodeling of the peptidoglycan of the DAP-R strain CB1664 has already started in exponential phase, becoming more extensive later during stationary growth phase. Similar to the report of Zhou and Cegelski for an MSSA strain [30], we observed in our DAP-R study strain, CB1664, an increase in the monomeric muropeptide Penta(Gln) (peak 3) and in muropeptides with an alanine, instead of glycine, in the interpeptide bridge (peaks 1, 7, and 8). We, therefore, suggest that the DAP-R strain modifies its CW by the incorporation of alanine, which leads to a reduced cross-linking of peptidoglycan. These changes are not present in the other strain pairs. Interestingly, we saw a notable decrease in the cyclic dimeric muropeptide (Tetra(Gln) Gly_5_-Tetra(Gln) Gly_5_), previously noted to be increased in a β-lactam-resistant strain [37]. While the increase in certain monomers can be reversed by the addition of glycine to the growth medium, the decrease of the cyclic peak cannot, indicating that these two events have different causes. As the increased monomeric peaks only appeared in stationary growth phase (i.e. glycine limited conditions [30]), one could speculate that the DAP-R strain CB1664 buffers stem peptide-containing muropeptides (peak 3: Penta(Gln), peak 5: Penta(Gln) Gly and peak 7: Penta(Gln) Ala)) and alanine until glycine becomes available again.

We tested the effect of additional glycine on the MIC against DAP, but saw no differences compared to normal medium (data not shown). This indicates that the remodeling of the peptidoglycan has no influence on DAP-R in the strain-pair CB1663/CB1664.

When we analyzed the amino acid composition of the peptidoglycan of strain pair CB1663/CB1664 after 24 h growth, we noted an increased alanine and glycine content for the DAP-R strains. This finding fits with the proposed monomeric muropeptide structures, which were increased. We did not observe them in another set of DAP-R clinical isolates [21].

Taken together, we provide new evidence here for the fact that an increase in CW thickness, as a consequence of an increased WTA content, and increased WTA D-alanylation is a relatively common phenotype amongst DAP-R S*. aureus* strains (including both MSSA [17] and MRSA). These phenotypic alterations are consistent with both observed changes in the positive surface charge characteristics and transcriptional enhancement of expression profiles of genes involved in the above CW phenotypes. Lastly, it appears clear that, in addition to a plethora of CM adaptations, well-defined perturbations of CW structural and functional metrics contribute to the DAP-R phenotype in *S. aureus*.

## Supporting Information

Figure S1 Cell wall biosynthesis. Peptidoglycan biosynthesis starts in the cytoplasm with the step-wise assembly of the precursor UDP-MurNAc-pentapeptide. This precursor is then added to undecaprenol-phosphate at the cytoplasmic membrane, resulting in Lipid I. The addition of GlcNAc from UDP-Glc*N*Ac forms Lipid II. In staphylocci, five glycine-residues from tRNAs are added before Lipid II is finally flipped over the cytoplasmic membrane by a yet unknown enzyme. Outside the cell, Lipid II is incorporated into the existing cell wall by the transpeptidase and transglycosylase reactions of penicillin-binding proteins (PBPs). WTA biosynthesis occurs directly at the cytoplasmic membrane, starting with the addition of GlcNAc-P from UDP-Glc*N*Ac to undecaprenol-phosphate (bracket). After the addition of Man*N*Ac the anchor structure is finished by adding 3 glycerol-P molecules. Then up to 40 ribitol-P molecules are polymerized step-wise until the WTA molecule is completed and finally transported across the CM by TagGH. The mature polymer is linked to the C6 atom of MurNAc in the peptidoglycan by a yet unidentified enzyme and then modified with GlcNAc and D-alanine (circled) (A). The organisation of WTA biosynthesis genes (B).

Figure S2 Expression profiles of *dltA* and *tagA* for strains CB5088/CB5098. Values from exponential (A) and stationary (B) growth phase RNA samples were normalized vs. housekeeping gene, *gyrB*, expression levels; data from the DAP-S strains were set to 1 to allow comparison of data from different samples with their respective DAP-R isolates.

Figure S3 Muropeptide pattern by HPLC analysis. The CW was isolated at OD_578_=0.7. The peptidoglycan was digested by the muraminidase mutanolysin and analyzed by HPLC. The overall muropeptide pattern of all strains was typical for *Staphylococcus aureus*. However, DAP-R strain CB1664 showed an increase in certain monomeric muropeptides vs. its respective DAP-S isolate (CB1663), which was not seen in the other three strain pairs.

Figure S4 Muropeptide structures. (**A**) Muropeptides are the subunits of the bacterial CW. The glycan part consists of N-acetylglucosamine (G) linked by a β-1,4 glycosidic bond to N-acetylmuramic acid (M). A polymer of these disaccharides forms the glycan backbone of the CW. Attached to M is the stem peptide (L-Ala – D-Gln – L-Lys – D-Ala – D-Ala). Added to the ε-amino group of L-Lys is the interpeptide bridge, which mainly consists of five Gly residues. The first Gly is sometimes seen to be replaced by Ala [29] and the second one by Ser [40]. Some muropeptides also contain Gly residues attached to the D-Ala on position four. They persist from former cross-links between two adjacent peptides from two different glycan strands. The peptide parts of the CW are indirectly cross-linked by the interpeptide bridge, forming a bond between the D-Ala on position four of the donor peptide and the fifth Gly of the interpeptide bridge of the adjacent stem peptide. Thereby, the terminal D-Ala of the donor peptide is cleaved off. Part (**B**) gives two examples of dimeric muropeptides. The upper part shows a classical Penta-Tetra dimer coming from two cross-linked glycan chains. Cross-linking in *S. aureus* can result in bigger muropeptides (e.g. trimers, tetramers,…) The bottom part shows the unique cyclic dimer with a double cross-link between two stem peptides [39].

Figure S5 Muropeptide analyses at different time points with the addition of glycine or alanine to the medium. We analyzed the muropeptide pattern of strain set CB1663/CB1664 at different time points, and tested whether the addition of glycine or alanine (~8 times the normal amount) to the medium had any effect. The peaks of strain CB1663 and CB1664 after 24h without the addition of extra amino acids were collected and analyzed by mass spectrometry (MS). The peaks at OD_578_=0.7 were labeled according to the retention time at 24 hrs.

Table S1 Muropeptide composition.

Table S2 Relative amounts of amino acids.

## References

[1] Liu C, Bayer A, Cosgrove SE, Daum RS, Fridkin SK et al. (2011) Clinical practice guidelines by the infectious diseases society of america for the treatment of methicillin-resistant *Staphylococcus aureus* infections in adults and children: executive summary. Clin Infect Dis 52: 285-292. doi:10.1093/cid/cir034. PubMed: 21217178. 21217178 10.1093/cid/cir034

[2] Howden BP, Davies JK, Johnson PD, Stinear TP, Grayson ML (2010) Reduced vancomycin susceptibility in *Staphylococcus aureus*, including vancomycin-intermediate and heterogeneous vancomycin-intermediate strains: resistance mechanisms, laboratory detection, and clinical implications. Clin Microbiol Rev 23: 99-139. doi:10.1128/CMR.00042-09. PubMed: 20065327. 20065327 10.1128/CMR.00042-09 PMC2806658

[3] Sakoulas G, Eliopoulos GM, Moellering RC Jr., Novick RP, Venkataraman L et al. (2003) *Staphylococcus aureus* accessory gene regulator (agr) group II: is there a relationship to the development of intermediate-level glycopeptide resistance? J Infect Dis 187: 929-938. doi:10.1086/368128. PubMed: 12660939. 12660939 10.1086/368128

[4] Wootton M, MacGowan AP, Walsh TR (2006) Comparative bactericidal activities of daptomycin and vancomycin against glycopeptide-intermediate *Staphylococcus aureus* (GISA) and heterogeneous GISA isolates. Antimicrob Agents Chemother 50: 4195-4197. doi:10.1128/AAC.00678-06. PubMed: 17043121. 17043121 10.1128/AAC.00678-06 PMC1693982

[5] Boucher HW, Sakoulas G (2007) Perspectives on Daptomycin resistance, with emphasis on resistance in *Staphylococcus aureus* . Clin Infect Dis 45: 601-608. doi:10.1086/520655. PubMed: 17682996. 17682996 10.1086/520655

[6] Hayden MK, Rezai K, Hayes RA, Lolans K, Quinn JP et al. (2005) Development of Daptomycin resistance in vivo in methicillin-resistant *Staphylococcus aureus* . J Clin Microbiol 43: 5285-5287. doi:10.1128/JCM.43.10.5285-5287.2005. PubMed: 16207998. 16207998 10.1128/JCM.43.10.5285-5287.2005 PMC1248493

[7] Skiest DJ (2006) Treatment failure resulting from resistance of *Staphylococcus aureus* to daptomycin. J Clin Microbiol 44: 655-656. doi:10.1128/JCM.44.2.655-656.2006. PubMed: 16455939. 16455939 10.1128/JCM.44.2.655-656.2006 PMC1392696

[8] Yang SJ, Kreiswirth BN, Sakoulas G, Yeaman MR, Xiong YQ et al. (2009) Enhanced expression of dltABCD is associated with the development of daptomycin nonsusceptibility in a clinical endocarditis isolate of *Staphylococcus aureus* . J Infect Dis 200: 1916-1920. doi:10.1086/648473. PubMed: 19919306. 19919306 10.1086/648473 PMC2779839

[9] Yang SJ, Nast CC, Mishra NN, Yeaman MR, Fey PD et al. (2010) Cell wall thickening is not a universal accompaniment of the daptomycin nonsusceptibility phenotype in *Staphylococcus aureus*: evidence for multiple resistance mechanisms. Antimicrob Agents Chemother 54: 3079-3085. doi:10.1128/AAC.00122-10. PubMed: 20498310. 20498310 10.1128/AAC.00122-10 PMC2916340

[10] Yang SJ, Xiong YQ, Dunman PM, Schrenzel J, François P et al. (2009) Regulation of mprF in daptomycin-nonsusceptible *Staphylococcus aureus* strains. Antimicrob Agents Chemother 53: 2636-2637. doi:10.1128/AAC.01415-08. PubMed: 19289517. 19289517 10.1128/AAC.01415-08 PMC2687189

[11] Bayer AS, Schneider T, Sahl HG (2013) Mechanisms of daptomycin resistance in *Staphylococcus aureus*: role of the cell membrane and cell wall. Ann N Y Acad Sci, 1277: 139–58. PubMed: 23215859. 23215859 10.1111/j.1749-6632.2012.06819.x PMC3556211

[12] Jones T, Yeaman MR, Sakoulas G, Yang SJ, Proctor RA et al. (2008) Failures in clinical treatment of *Staphylococcus aureus* Infection with daptomycin are associated with alterations in surface charge, membrane phospholipid asymmetry, and drug binding. Antimicrob Agents Chemother 52: 269-278. doi:10.1128/AAC.00719-07. PubMed: 17954690. 17954690 10.1128/AAC.00719-07 PMC2223911

[13] Mishra NN, Yang SJ, Sawa A, Rubio A, Nast CC et al. (2009) Analysis of cell membrane characteristics of in vitro-selected daptomycin-resistant strains of methicillin-resistant *Staphylococcus aureus* . Antimicrob Agents Chemother 53: 2312-2318. doi:10.1128/AAC.01682-08. PubMed: 19332678. 19332678 10.1128/AAC.01682-08 PMC2687258

[14] Mishra NN, McKinnell J, Yeaman MR, Rubio A, Nast CC et al. (2011) In vitro cross-resistance to daptomycin and host defense cationic antimicrobial peptides in clinical methicillin-resistant *Staphylococcus aureus* isolates. Antimicrob Agents Chemother 55: 4012-4018. doi:10.1128/AAC.00223-11. PubMed: 21709105. 21709105 10.1128/AAC.00223-11 PMC3165344

[15] Julian K, Kosowska-Shick K, Whitener C, Roos M, Labischinski H et al. (2007) Characterization of a daptomycin-nonsusceptible vancomycin-intermediate *Staphylococcus aureus* strain in a patient with endocarditis. Antimicrob Agents Chemother 51: 3445-3448. doi:10.1128/AAC.00559-07. PubMed: 17620372. 17620372 10.1128/AAC.00559-07 PMC2043240

[16] Pillai SK, Gold HS, Sakoulas G, Wennersten C, Moellering RC Jr. et al. (2007) Daptomycin nonsusceptibility in *Staphylococcus aureus* with reduced vancomycin susceptibility is independent of alterations in MprF. Antimicrob Agents Chemother 51: 2223-2225. doi:10.1128/AAC.00202-07. PubMed: 17404001. 17404001 10.1128/AAC.00202-07 PMC1891363

[17] Peschel A, Jack RW, Otto M, Collins LV, Staubitz P et al. (2001) *Staphylococcus aureus* resistance to human defensins and evasion of neutrophil killing via the novel virulence factor MprF is based on modification of membrane lipids with l-lysine. J Exp Med 193: 1067-1076. doi:10.1084/jem.193.9.1067. PubMed: 11342591. 11342591 10.1084/jem.193.9.1067 PMC2193429

[18] Ernst CM, Peschel A (2011) Broad-spectrum antimicrobial peptide resistance by MprF-mediated aminoacylation and flipping of phospholipids. Mol Microbiol 80: 290-299. doi:10.1111/j.1365-2958.2011.07576.x. PubMed: 21306448. 21306448 10.1111/j.1365-2958.2011.07576.x

[19] Mohedano ML, Overweg K, de la Fuente A, Reuter M, Altabe S et al. (2005) Evidence that the essential response regulator YycF in Streptococcus pneumoniae modulates expression of fatty acid biosynthesis genes and alters membrane composition. J Bacteriol 187: 2357-2367. doi:10.1128/JB.187.7.2357-2367.2005. PubMed: 15774879. 15774879 10.1128/JB.187.7.2357-2367.2005 PMC1065234

[20] Dubrac S, Bisicchia P, Devine KM, Msadek T (2008) A matter of life and death: cell wall homeostasis and the WalKR (YycGF) essential signal transduction pathway. Mol Microbiol 70: 1307-1322. doi:10.1111/j.1365-2958.2008.06483.x. PubMed: 19019149. 19019149 10.1111/j.1365-2958.2008.06483.x

[21] Bertsche U, Weidenmaier C, Kuehner D, Yang SJ, Baur S et al. (2011) Correlation of Daptomycin-Resistance in a Clinical *Staphylococcus aureus* Strain with Increased Cell Wall Teichoic Acid Production and D-alanylation. Antimicrob Agents Chemother. 10.1128/AAC.01226-10 PMC3147621 21606222

[22] Ginsberg C, Zhang YH, Yuan Y, Walker S (2006) In vitro reconstitution of two essential steps in wall teichoic acid biosynthesis. ACS Chem Biol 1: 25-28. doi:10.1021/cb0500041. PubMed: 17163636. 17163636 10.1021/cb0500041

[23] Xia G, Peschel A (2008) Toward the pathway of S. aureus WTA biosynthesis. Chem Biol 15: 95-96. doi:10.1016/j.chembiol.2008.02.005. PubMed: 18291312. 18291312 10.1016/j.chembiol.2008.02.005

[24] Peschel A, Otto M, Jack RW, Kalbacher H, Jung G et al. (1999) Inactivation of the dlt operon in *Staphylococcus aureus* confers sensitivity to defensins, protegrins, and other antimicrobial peptides. J Biol Chem 274: 8405-8410. doi:10.1074/jbc.274.13.8405. PubMed: 10085071. 10085071 10.1074/jbc.274.13.8405

[25] Weidenmaier C, Kokai-Kun JF, Kristian SA, Chanturiya T, Kalbacher H et al. (2004) Role of teichoic acids in *Staphylococcus aureus* nasal colonization, a major risk factor in nosocomial infections. Nat Med 10: 243-245. doi:10.1038/nm991. PubMed: 14758355. 14758355 10.1038/nm991

[26] Kristian SA, Datta V, Weidenmaier C, Kansal R, Fedtke I et al. (2005) D-alanylation of teichoic acids promotes group a streptococcus antimicrobial peptide resistance, neutrophil survival, and epithelial cell invasion. J Bacteriol 187: 6719-6725. doi:10.1128/JB.187.19.6719-6725.2005. PubMed: 16166534. 16166534 10.1128/JB.187.19.6719-6725.2005 PMC1251589

[27] Peschel A, Ottenwälder B, Götz F (1996) Inducible production and cellular location of the epidermin biosynthetic enzyme EpiB using an improved staphylococcal expression system. FEMS Microbiol Lett 137: 279-284. doi:10.1111/j.1574-6968.1996.tb08119.x. PubMed: 8998998. 8998998 10.1111/j.1574-6968.1996.tb08119.x

[28] Peschel A, Vuong C, Otto M, Götz F (2000) The D-alanine residues of *Staphylococcus aureus* teichoic acids alter the susceptibility to vancomycin and the activity of autolytic enzymes. Antimicrob Agents Chemother 44: 2845-2847. doi:10.1128/AAC.44.10.2845-2847.2000. PubMed: 10991869. 10991869 10.1128/aac.44.10.2845-2847.2000 PMC90160

[29] de Jonge BL, Chang YS, Gage D, Tomasz A (1992) Peptidoglycan composition of a highly methicillin-resistant Staphylococcus aureus strain. The role of penicillin binding protein 2A. J Biol Chem 267: 11248-11254. 1597460

[30] Zhou X, Cegelski L (2012) Nutrient-Dependent Structural Changes in S. aureus Peptidoglycan Revealed by Solid-State NMR Spectroscopy. Biochemistry 51: 8143-8153. doi:10.1021/bi3012115. PubMed: 22974326. 22974326 10.1021/bi3012115 PMC3554850

[31] Camargo IL, Neoh HM, Cui L, Hiramatsu K (2008) Serial daptomycin selection generates daptomycin-nonsusceptible *Staphylococcus aureus* strains with a heterogeneous vancomycin-intermediate phenotype. Antimicrob Agents Chemother 52: 4289-4299. doi:10.1128/AAC.00417-08. PubMed: 18824611. 18824611 10.1128/AAC.00417-08 PMC2592861

[32] Cui L, Tominaga E, Neoh HM, Hiramatsu K (2006) Correlation between Reduced Daptomycin Susceptibility and Vancomycin Resistance in Vancomycin-Intermediate *Staphylococcus aureus* . Antimicrob Agents Chemother 50: 1079-1082. doi:10.1128/AAC.50.3.1079-1082.2006. PubMed: 16495273. 16495273 10.1128/AAC.50.3.1079-1082.2006 PMC1426436

[33] McAleese F, Wu SW, Sieradzki K, Dunman P, Murphy E et al. (2006) Overexpression of genes of the cell wall stimulon in clinical isolates of *Staphylococcus aureus* exhibiting vancomycin-intermediate- S. aureus-type resistance to vancomycin. J Bacteriol 188: 1120-1133. doi:10.1128/JB.188.3.1120-1133.2006. PubMed: 16428416. 16428416 10.1128/JB.188.3.1120-1133.2006 PMC1347359

[34] Muthaiyan A, Silverman JA, Jayaswal RK, Wilkinson BJ (2008) Transcriptional profiling reveals that daptomycin induces the *Staphylococcus aureus* cell wall stress stimulon and genes responsive to membrane depolarization. Antimicrob Agents Chemother 52: 980-990. doi:10.1128/AAC.01121-07. PubMed: 18086846. 18086846 10.1128/AAC.01121-07 PMC2258546

[35] Boyle-Vavra S, Jones M, Gourley BL, Holmes M, Ruf R et al. (2011) Comparative genome sequencing of an isogenic pair of USA800 clinical methicillin-resistant *Staphylococcus aureus* isolates obtained before and after daptomycin treatment failure. Antimicrob Agents Chemother 55: 2018-2025. doi:10.1128/AAC.01593-10. PubMed: 21343446. 21343446 10.1128/AAC.01593-10 PMC3088213

[36] Fischer A, Yang SJ, Bayer AS, Vaezzadeh AR, Herzig S et al. (2011) Daptomycin resistance mechanisms in clinically derived *Staphylococcus aureus* strains assessed by a combined transcriptomics and proteomics approach. J Antimicrob Chemother 66: 1696-1711. doi:10.1093/jac/dkr195. PubMed: 21622973. 21622973 10.1093/jac/dkr195 PMC3133485

[37] Göhring N, Fedtke I, Xia G, Jorge AM, Pinho MG et al. (2011) New Role of the Disulfide Stress Effector YjbH in β-Lactam Susceptibility of *Staphylococcus aureus* . Antimicrob Agents Chemother 55: 5452-5458. PubMed: 21947404. 21947404 10.1128/AAC.00286-11 PMC3232775

[38] Strandén AM, Ehlert K, Labischinski H, Berger-Bächi B (1997) Cell wall monoglycine cross-bridges and methicillin hypersusceptibility in a femAB null mutant of methicillin-resistant *Staphylococcus aureus* . J Bacteriol 179: 9-16. PubMed: 8981974. 8981974 10.1128/jb.179.1.9-16.1997 PMC178655

[39] Boneca IG, Xu N, Gage DA, de Jonge BL, Tomasz A (1997) Structural characterization of an abnormally cross-linked muropeptide dimer that is accumulated in the peptidoglycan of methicillin- and cefotaxime-resistant mutants of *Staphylococcus aureus* . J Biol Chem 272: 29053-29059. doi:10.1074/jbc.272.46.29053. PubMed: 9360979. 9360979 10.1074/jbc.272.46.29053

[40] de Jonge BL, Sidow T, Chang YS, Labischinski H, Berger-Bachi B et al. (1993) Altered muropeptide composition in *Staphylococcus aureus* strains with an inactivated femA locus. J Bacteriol 175: 2779-2782. PubMed: 8478340. 8478340 10.1128/jb.175.9.2779-2782.1993 PMC204585

