# Supplementary figures and images for: Increased Cell Wall Teichoic Acid Production and D-alanylation Are Common Phenotypes among Daptomycin-Resistant Methicillin-Resistant Staphylococcus aureus (MRSA) Clinical Isolates
Source: PLoS One. 2013 Jun 13;8(6):e67398. doi: 10.1371/journal.pone.0067398 (PMC3681945; doi:10.1371/journal.pone.0067398)

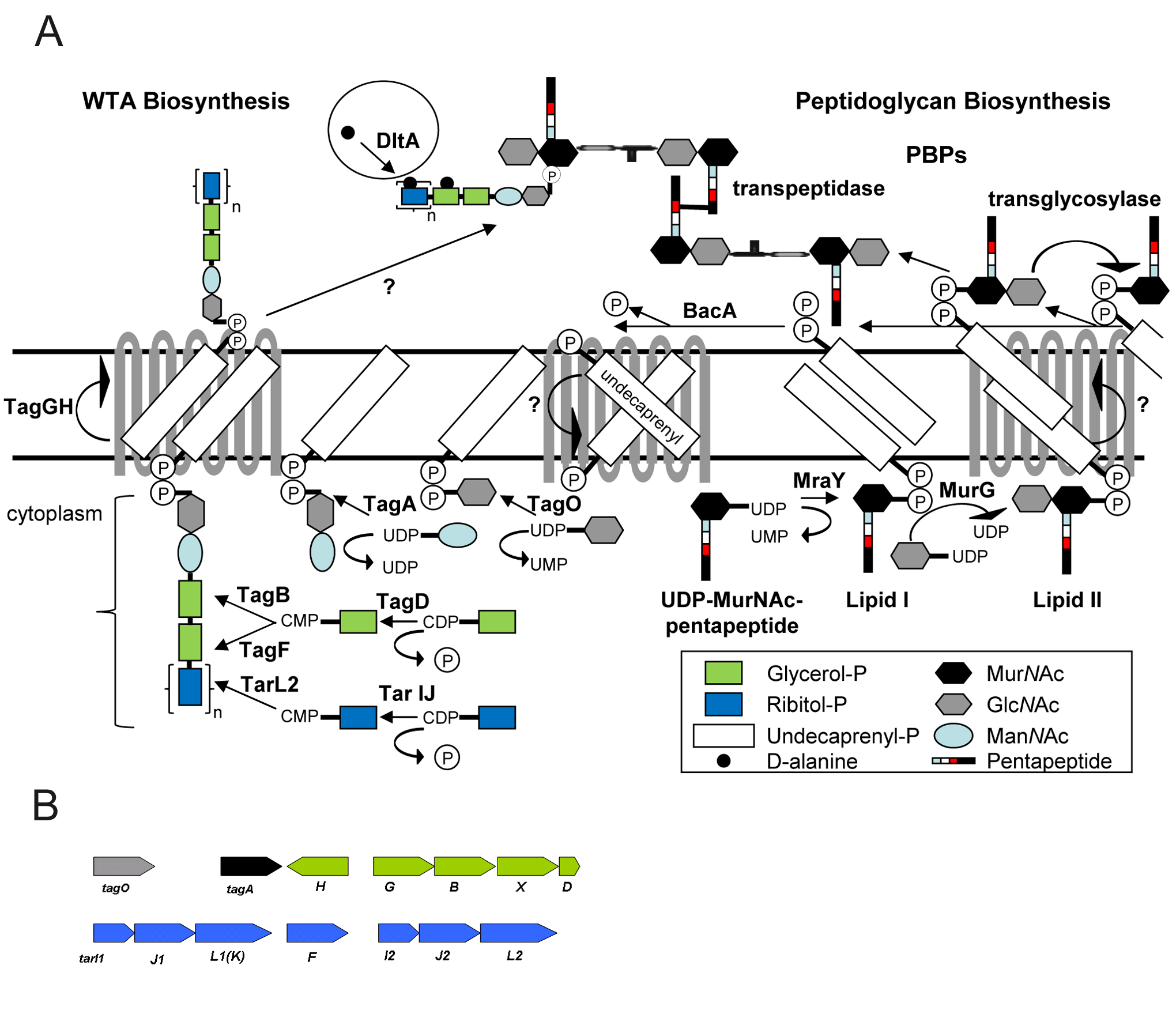

Supplement: Figure S1 — Peptidoglycan biosynthesis starts in the cytoplasm with the step-wise assembly of the precursor UDP-MurNAc-pentapeptide. This precursor is then added to undecaprenol-phosphate at the cytoplasmic membrane, resulting in Lipid I. The addition of GlcNAc from UDP-GlcNAc forms Lipid II. In staphylocci, five glycine-residues from tRNAs are added before Lipid II is finally flipped over the cytoplasmic membrane by a yet unknown enzyme. Outside the cell, Lipid II is incorporated into the existing cell wall by the transpeptidase and transglycosylase reactions of penicillin-binding proteins (PBPs). WTA biosynthesis occurs directly at the cytoplasmic membrane, starting with the addition of GlcNAc-P from UDP-GlcNAc to undecaprenol-phosphate (bracket). After the addition of ManNAc the anchor structure is finished by adding 3 glycerol-P molecules. Then up to 40 ribitol-P molecules are polymerized step-wise until the WTA molecule is completed and finally transported across the CM by TagGH. The mature polymer is linked to the C6 atom of MurNAc in the peptidoglycan by a yet unidentified enzyme and then modified with GlcNAc and D-alanine (circled) (A). The organisation of WTA biosynthesis genes (B). [file pone.0067398.s001.tif]

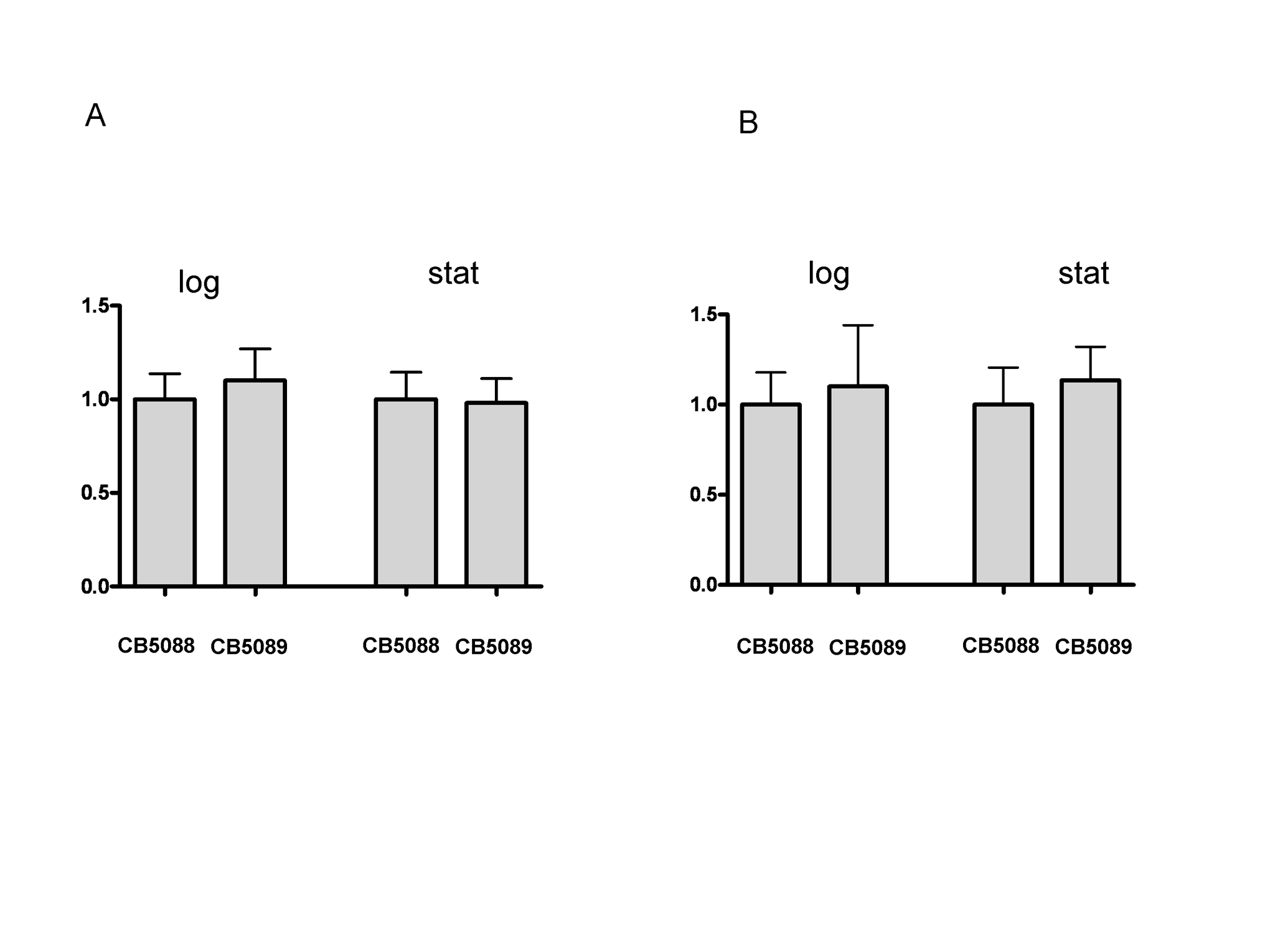

Supplement: Figure S2 — Values from exponential (A) and stationary (B) growth phase RNA samples were normalized vs. housekeeping gene, gyrB, expression levels; data from the DAP-S strains were set to 1 to allow comparison of data from different samples with their respective DAP-R isolates. [file pone.0067398.s002.tif]

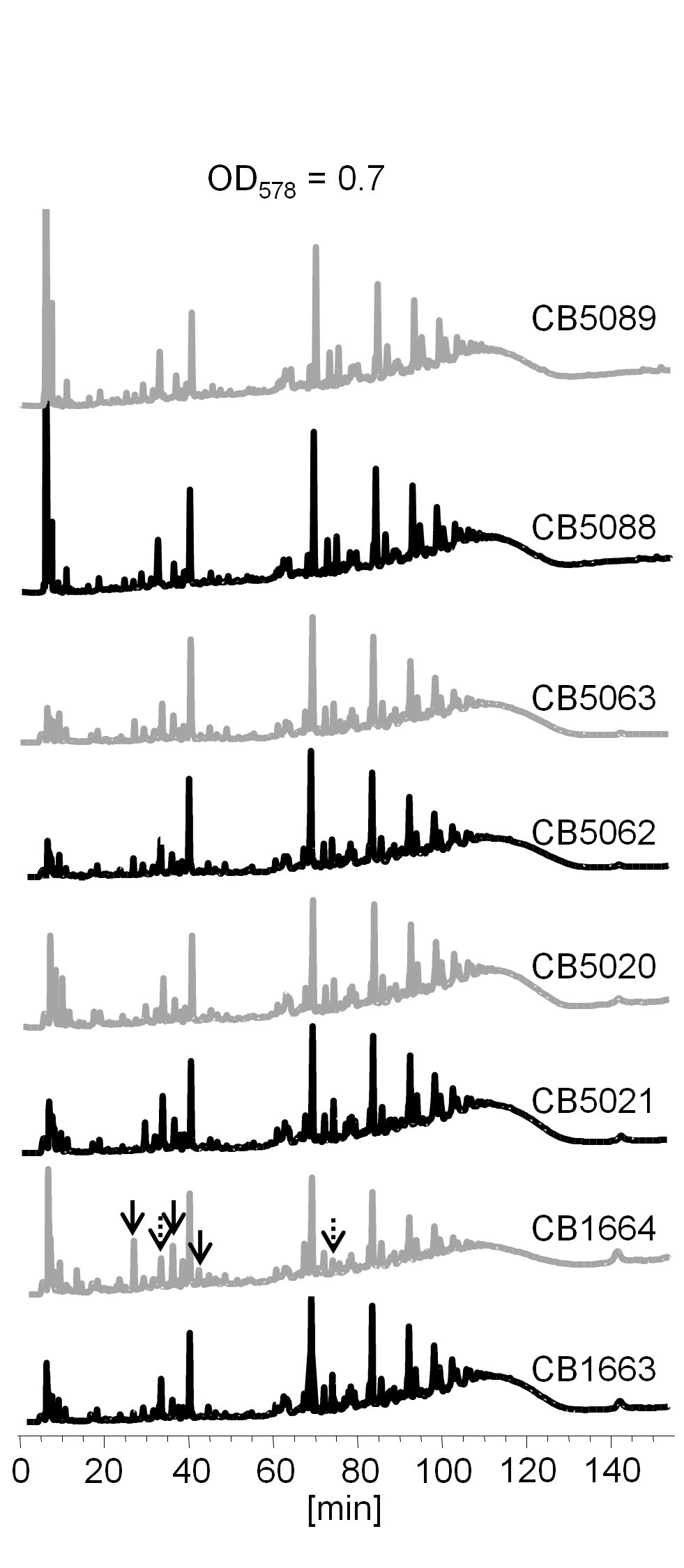

Supplement: Figure S3 — The CW was isolated at OD578=0.7. The peptidoglycan was digested by the muraminidase mutanolysin and analyzed by HPLC. The overall muropeptide pattern of all strains was typical for Staphylococcus aureus. However, DAP-R strain CB1664 showed an increase in certain monomeric muropeptides vs. its respective DAP-S isolate (CB1663), which was not seen in the other three strain pairs. [file pone.0067398.s003.tif]

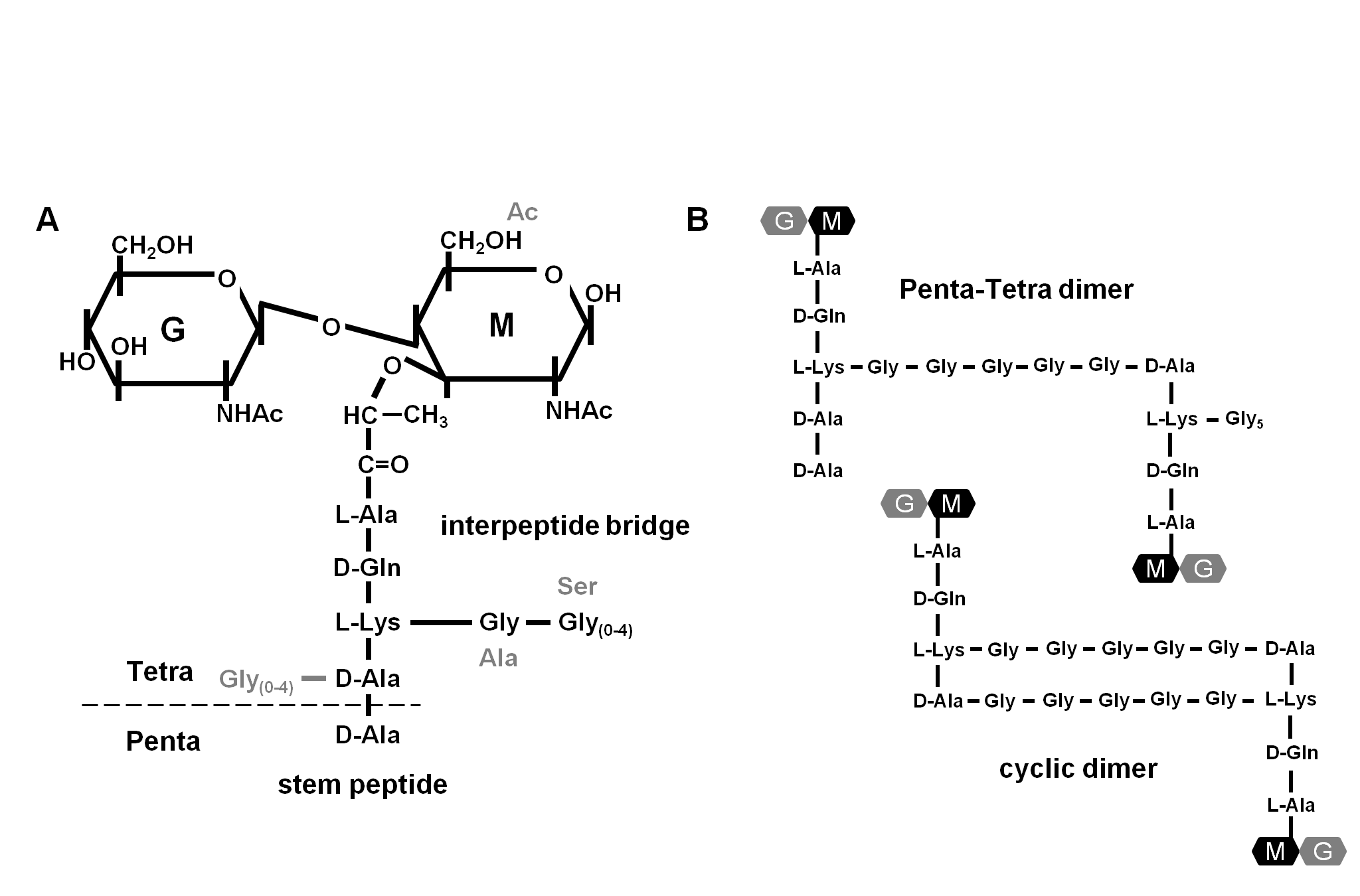

Supplement: Figure S4 — (A) Muropeptides are the subunits of the bacterial CW. The glycan part consists of N-acetylglucosamine (G) linked by a β-1,4 glycosidic bond to N-acetylmuramic acid (M). A polymer of these disaccharides forms the glycan backbone of the CW. Attached to M is the stem peptide (L-Ala – D-Gln – L-Lys – D-Ala – D-Ala). Added to the ε-amino group of L-Lys is the interpeptide bridge, which mainly consists of five Gly residues. The first Gly is sometimes seen to be replaced by Ala [29] and the second one by Ser [40]. Some muropeptides also contain Gly residues attached to the D-Ala on position four. They persist from former cross-links between two adjacent peptides from two different glycan strands. The peptide parts of the CW are indirectly cross-linked by the interpeptide bridge, forming a bond between the D-Ala on position four of the donor peptide and the fifth Gly of the interpeptide bridge of the adjacent stem peptide. Thereby, the terminal D-Ala of the donor peptide is cleaved off. Part (B) gives two examples of dimeric muropeptides. The upper part shows a classical Penta-Tetra dimer coming from two cross-linked glycan chains. Cross-linking in S. aureus can result in bigger muropeptides (e.g. trimers, tetramers,…) The bottom part shows the unique cyclic dimer with a double cross-link between two stem peptides [39]. [file pone.0067398.s004.tif]

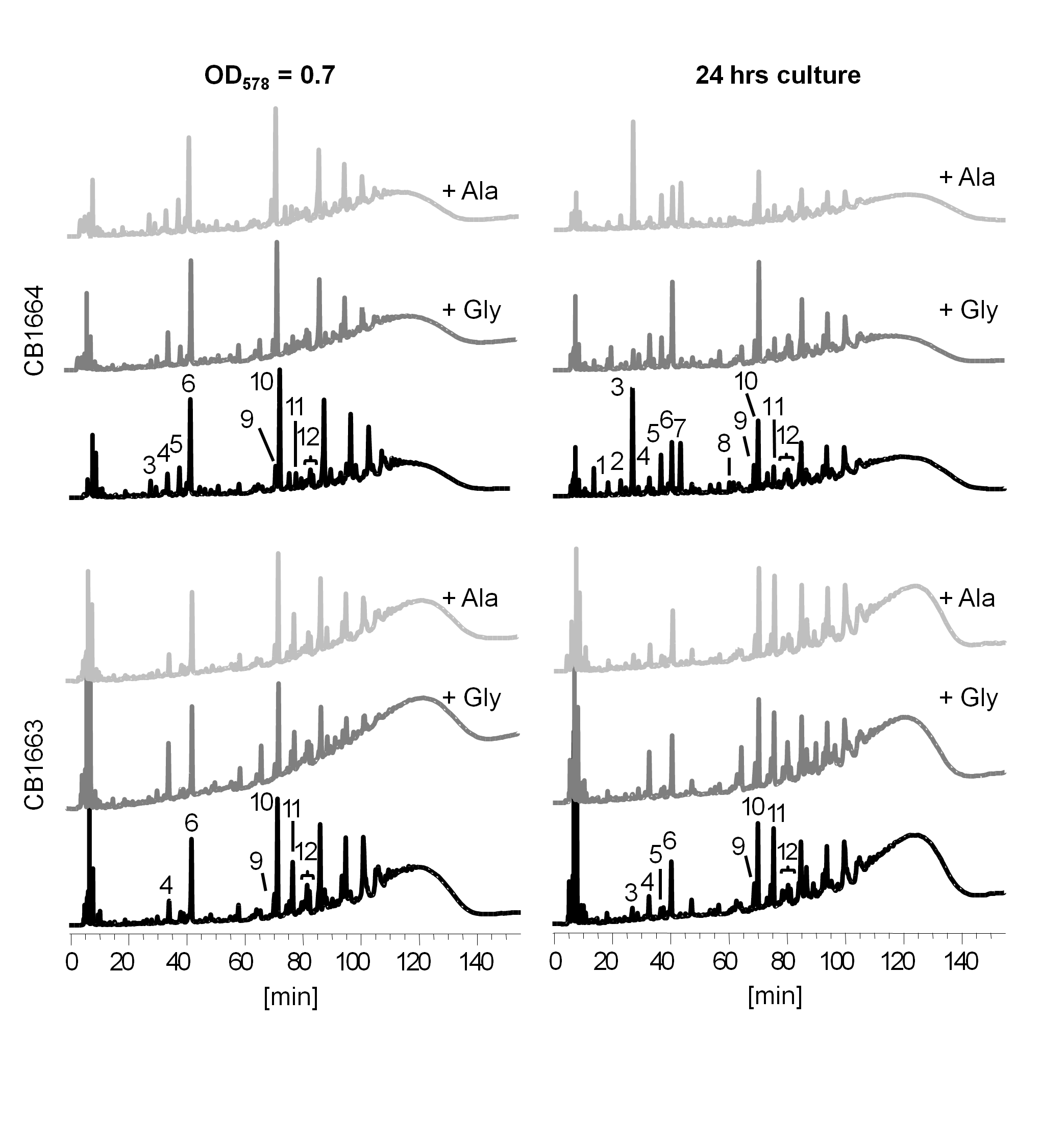

Supplement: Figure S5 — We analyzed the muropeptide pattern of strain set CB1663/CB1664 at different time points, and tested whether the addition of glycine or alanine (~8 times the normal amount) to the medium had any effect. The peaks of strain CB1663 and CB1664 after 24h without the addition of extra amino acids were collected and analyzed by mass spectrometry (MS). The peaks at OD578=0.7 were labeled according to the retention time at 24 hrs. [file pone.0067398.s005.tif]
