# Supplementary material for: Increased Cell Wall Teichoic Acid Production and D-alanylation Are Common Phenotypes among Daptomycin-Resistant Methicillin-Resistant Staphylococcus aureus (MRSA) Clinical Isolates
Source: PLoS One. 2013 Jun 13;8(6):e67398. doi: 10.1371/journal.pone.0067398 (PMC3681945; doi:10.1371/journal.pone.0067398)
Supplement: Table S1 [file pone.0067398.s006.doc]

**Table S**1: Muropeptide composition

| Peak | proposed muropeptide | neutral mass determined | neutral mass calculated | amount at OD_578_=0.7 in % | | amount at  24 hrs in % | |
| --- | --- | --- | --- | --- | --- | --- | --- |
|  |  |  |  | CB1663 | CB1664 | CB1663 | CB1664 |
|  |  |  |  |  |  |  |  |
| 1 | Tetra(Gln)AlaGly | 1024.6 | 1024.5 | n.d. | n.d. | n.d. | 0.305 |
| 2 | Tetra(Gln) | 896.4 | 896.4 | n.d. | n.d. | n.d. | 0.340 |
| 3 | Penta(Gln) | 967.5 | 967.5 | 0.097 | 0.436 | 0.253 | 2.475 |
| 4 | Tetra(Gln)Gly_6_ | 1238.6 | 1238.6 | 0.494 | 0.775 | 0.407 | 0.370 |
|  | Tetra(Gln)Gly_7_ | 1267.1 | 1267.1 |  |  |  |  |
|  | Tetra(Gln)Gly_8_ | 1295.6 | 1295.6 |  |  |  |  |
|  | Tetra(Gln)Gly_9_ | 1324.1 | 1324.1 |  |  |  |  |
| 5 | Penta(Gln)Gly | 1024 | 1024.5 | 0.238 | 0.921 | 0.192 | 0.918 |
| 6 | Penta(Gln)Gly_5_ | 1252.6 | 1252.6 | 1.670 | 3.070 | 0.901 | 1.215 |
| 7 | Penta(Gln)Ala | 1038.6 | 1038.5 | n.d. | n.d. | n.d. | 1.455 |
| 8 | acetylated Penta(Gln)Ala | 1080.6 | 1080.5 | n.d. | n.d. | n.d. | 0.344 |
| 9 | Penta(Gln)Gly_5_-Tetra(Gln)Gly | 2189.1 | 2188.9 | 0.866 | 1.423 | 0.597 | 0.888 |
| 10 | Penta(Gln)Gly_5_-Tetra(Gln)Gly_5_ | 2417.1 | 2417 | 2.458 | 3.830 | 1.340 | 1.645 |
| 11 | cyclic peak (Tetra(Gln)Gly_5_-Tetra(Gln)Gly_5_) | 2328 | 2328 | 1.429 | 0.710 | 1.375 | 0.605 |
| 12 | Penta(Gln)SerGly_4_-Tetra(Gln)Ala | 2231.1 | 2231 | 3.014 | 3.260 | 2.247 | 2.587 |
|  | acetylated Penta(Gln)Gly_5_-Tetra(Gln)Gly_5_ | 2459.1 | 2459 |  |  |  |  |

Muropeptides 1 to 12 were analyzed by mass spectrometry. The determined neutral mass is given for each compound and a structure was proposed according to calculated masses and to known retention times of muropeptides [29,39]. The relative amount of each muropeptide was calculated as percentage of all muropeptides (n.d.: not detectable).
