# Supplementary material for: Increased Cell Wall Teichoic Acid Production and D-alanylation Are Common Phenotypes among Daptomycin-Resistant Methicillin-Resistant Staphylococcus aureus (MRSA) Clinical Isolates
Source: PLoS One. 2013 Jun 13;8(6):e67398. doi: 10.1371/journal.pone.0067398 (PMC3681945; doi:10.1371/journal.pone.0067398)
Supplement: Table S2 [file pone.0067398.s007.doc]

Table S2: Relative amounts of amino acids

|  | Glx | Gly | Ala | Lys |
| --- | --- | --- | --- | --- |
|  |  |  |  |  |
| CB1663 | 1.0 ± 0.0 | 6.2 ± 1.6 | 3.8 ± 1.0 | 1.0 ± 0.2 |
| CB1664 | 1.0 ± 0.0 | 12.2 ± 3.1* | 7.3 ± 1.4* | 2.3 ± 0.3** |

The peptidoglycan isolated after 24 hrs was hydrolyzed by HCl and the amino acids were analyzed by HPLC using precolumn derivatization by *o*-phthaldialdehyd (OPA). It cannot be distinguished between glutamate and glutamine, as glutamine is converted into glutamate during hydrolysis. Therefore, the amount of both residues was combined as Glx and set to 1.0. n=3. Statistical analysis was performed by Student’s t-test. Significance: * p-value < 0.05 vs parental strain; ** p-value < 0.001 vs parental strain.
